# Supplementary material for: MetaRibo-Seq measures translation in microbiomes
Source: Nat Commun. 2020 Jun 29;11:3268. doi: 10.1038/s41467-020-17081-z (PMC7324362; doi:10.1038/s41467-020-17081-z)
Supplement: Supplementary file 10 — Supplementary Data 7 [file 41467_2020_17081_MOESM10_ESM.zip › File2/Confidence_High_Taxonomy/57889_out.krona.html]

Javascript must be enabled to view this page.

members
magnitude
magnitudeUnassigned
count
unassigned
taxon
rank

57889\_out

18

18
2
superkingdom

18
1239
phylum

186801
18
class

order
186802
18

family
541000
18

genus
18
1263


SRS1041038\_contig\_number\_8479SRS143372\_contig\_number\_6925
species
2293169
2

species

SRS017103\_contig\_number\_contig-100\_671.231542SRS024331\_contig\_number\_contig-100\_120.208695
2
1160721

species

SRS078242\_contig\_number\_contig-100\_2020.87354
1
2293178

species

SRS011302\_contig\_number\_25433SRS144297\_contig\_number\_12107
2
2293177

2293233
1

SRS077730\_contig\_number\_contig-100\_4340.4340
species

2293149
1

SRS144714\_contig\_number\_24782
species

species

SRS097958\_contig\_number\_16883
1
1262962

species

SRS013940\_contig\_number\_20067
1
2293156


SRS013638\_contig\_number\_13984SRS016495\_contig\_number\_2342SRS098061\_contig\_number\_106SRS1055049\_contig\_number\_1042SRS149075\_contig\_number\_16488
species
41978
5

species

SRS053335\_contig\_number\_18137SRS1041157\_contig\_number\_6797
2
165186
